# Cost‐effectiveness of dexamethasone and triamcinolone for the treatment of diabetic macular oedema in Finland: A Markov‐model

**DOI:** 10.1111/aos.14745

**Published:** 2021-01-09

**Authors:** Mari Pesonen, Eila Kankaanpää, Pasi Vottonen

**Affiliations:** ^1^ University of Eastern Finland Kuopio Finland; ^2^ Suomen Terveystalo Oy and University of Eastern Finland Kuopio Finland

**Keywords:** cost‐effectiveness, Markov‐modelling, diabetic macular oedema, dexamethasone, triamcinolone

## Abstract

**Purpose:**

Diabetic macular oedema (DMO), a complication of diabetes, causes vision loss and blindness. Corticosteroids are usually used as a second‐line treatment. The aim of this study was to analyse the cost‐effectiveness of dexamethasone implants compared to cheaper and more frequently applied triamcinolone injections.

**Methods:**

Markov‐modelling, which incorporated both eyes, was used for economic evaluation. The model consisted of five health states based on visual acuity, illustrating the progression of DMO. A cycle length of five months was chosen for dexamethasone and four months for triamcinolone. Time horizons of two and five years were applied. Transition probabilities and health state utilities were sourced from previous studies. The perspective used in this analysis was the hospital perspective. The health care costs were acquired from Kuopio University Hospital in Finland.

**Results:**

In this cost‐effectiveness analysis, the incremental cost‐effectiveness ratio ICER with 3% discount rate was €56 591/QALY for a two‐year follow‐up and −€1 110 942/QALY for a five‐year follow‐up. In order to consider dexamethasone as cost‐effective over a 2‐year time horizon, the WTP needs to be around €55 000/QALY. Over the five‐year follow‐up, triamcinolone is clearly a dominant treatment. Sensitivity analyses support the cost‐effectiveness of dexamethasone over a 2‐year time horizon.

**Conclusions:**

Since the sensitivity analyses support the results, dexamethasone would be a cost‐effective treatment during the first two years with WTP threshold around €55 000/QALY, and triamcinolone would be a convenient treatment after that. This recommendation is in line with the guidelines of EURETINA.

## Background

A continuously increasing number of diabetes mellitus (DM) patients has led to an increase in its complications, of which diabetic retinopathy (DR) is one of the most common (Zheng et al. 2012; Guariguata et al. 2014). Globally, DR accounts for about 2.6% of all vision losses (Leasher et al. 2016), and in Finland the percentage is slightly higher: 7% for the working‐age population and 3% for the elderly (Ojamo 2018). Diabetic macular oedema (DMO) is one form of diabetic retinopathy and it mainly accounts for vision loss and blindness, especially in the working‐age population (Miller & Fortun 2018). Although the incidence of diabetic retinopathy has decreased during the last few decades (Liew et al. 2017), nearly half of the patients do not achieve a balance with their DM (American Diabetes Association 2019), thus the risk of complications is still significant.

Pathogenesis of diabetic macular oedema is multifactorial. Both inflammatory mediators and vasogenic mediators, such as vascular endothelial growth factors (VEGFs), are activated and create disruption to the retina. As the inner blood‐retinal barrier breaks down, fluids and proteins leak into the retina. This leakage creates oedema of the macula and thereby decreases the retinal transparency (Stewart 2012; Duh et al. 2017; Miller & Fortun 2018).

Intravitreal medication is needed in order to prevent vision loss in DMO. Anti‐vascular endothelial growth factor (anti‐VEGF) injections (ranibizumab, aflibercept, and bevacizumab) are used as a first‐line treatment for DMO, whereas corticosteroids are possible as a second‐line treatment (Rajendran & Badole 2018; Virgili et al. 2018). Anti‐VEGF injections (ranibizumab) are proven to be cost‐effective compared to corticosteroids (triamcinolone) (Pershing et al. 2014), mainly because corticosteroids cause more adverse events than anti‐VEGF injections (Maturi et al. 2018; Mehta et al. 2018). In certain cases, such as poor compliance or contraindications, corticosteroids could be used as a first‐line treatment, too (Schmidt‐Erfurt et al. 2017; Rajendran & Badole 2018).

A need for different treatment options for anti‐VEGF treatment is clear because as many as 30–50% of patients do not respond to anti‐VEGF treatment properly (Duh et al. 2017; Shah et al. 2017). Corticosteroids have a beneficial impact on visual acuity, central macular thickness, and hard exudates, since they are able to control both inflammatory and vasogenic mediators (Lazic et al. 2014; Shin et al. 2017). Patients who do not respond to anti‐VEGF treatment should proceed to corticosteroids as soon as possible (Busch et al. 2018).

Possible choices for corticosteroid treatment are intravitreal triamcinolone injections and intravitreal dexamethasone or fluocinolone implants (Al‐Dhibi & Arevalo 2013; Schmidt‐Erfurt et al. 2017). For DMO, triamcinolone injections are used off‐label (Maniadakis & Konstantakopoulou 2019), and they have a short duration of action (Qi et al. 2012). Frequent injections of triamcinolone are required to maintain favourable effects (Dang et al. 2014). A dexamethasone implant enables the medicine to be released slowly and for a longer period of time, which can lead to better compliance as needed injections become rarer (Chang‐Lin et al. 2011; Miller & Fortun 2018). Fluocinolone implants have been withdrawn from the market in many countries, including Finland, due to safety issues (Kiddee et al. 2013; Finnish Medical Agency 2019).

Direct clinical comparisons between dexamethasone and triamcinolone have shown that dexamethasone and triamcinolone are somewhat equally effective although triamcinolone causes more adverse events, and re‐treatment is required sooner than with dexamethasone (Dang et al. 2014). Mylonas et al. (2017) compared dexamethasone and triamcinolone in cystic macular oedema and concluded that there was no significant difference in vision acuity improvement between the treatments, but that triamcinolone better reduced oedema of the macula. These treatments are also used for central or branch retinal vein occlusion in which triamcinolone is found to be more effective than dexamethasone (Smiddy 2011; Ford et al. 2014).

The frequency of injections affects the cost of the entire treatment and, in addition, unit costs of triamcinolone injections and dexamethasone implants differ significantly. As nowadays every medical treatment in use should be cost‐effective, there is a need to evaluate costs and effectiveness in the treatment of DMO. There are very few previous studies concerning the cost‐effectiveness of corticosteroids in the treatment of DMO. The cost‐effectiveness of corticosteroids is studied compared to anti‐VEGF injections and laser (Dewan et al. 2012; Pershing et al. 2014), and dexamethasone and fluocinolone are compared in pseudophakic eyes (Pochopien et al. 2019). It seems that to date, there are no cost‐effectiveness studies of triamcinolone injections compared to other corticosteroids for DMO nor the other macular oedemas. Cost‐effectiveness evaluations between corticosteroids alone are rare altogether.

The aim of this study was to compare the cost‐effectiveness of triamcinolone injections and dexamethasone implants. A Markov transition model, which incorporated both eyes, was adapted for this purpose. Corticosteroids were used as a first‐line treatment in the model, as this is also a clinically possible option. The same results are also applicable for corticosteroids as a second‐line treatment, since previous treatment does not seem to influence corticosteroid effectiveness (Malclés et al. 2017).

## Materials and methods

### The model

A Markov transition model can be used to evaluate diseases using certain health states combined with different costs and different utilities. A patient can be in one health state at a particular point in time and move on to another health state during a series of cycles. The result of the Markov model is an incremental cost‐effectiveness ratio (ICER), which compares the difference in costs between different treatments to their difference in effectiveness (Drummond et al. 2015). Quality Adjusted Life Years (QALYs) are used to represent the effectiveness in this model. It is important to incorporate both eyes in the model because it is possible, even likely, that only one eye is affected and the other one maintains good visual acuity. Both eyes are therefore considered when estimating costs and QALYs.

**Fig. 1:**
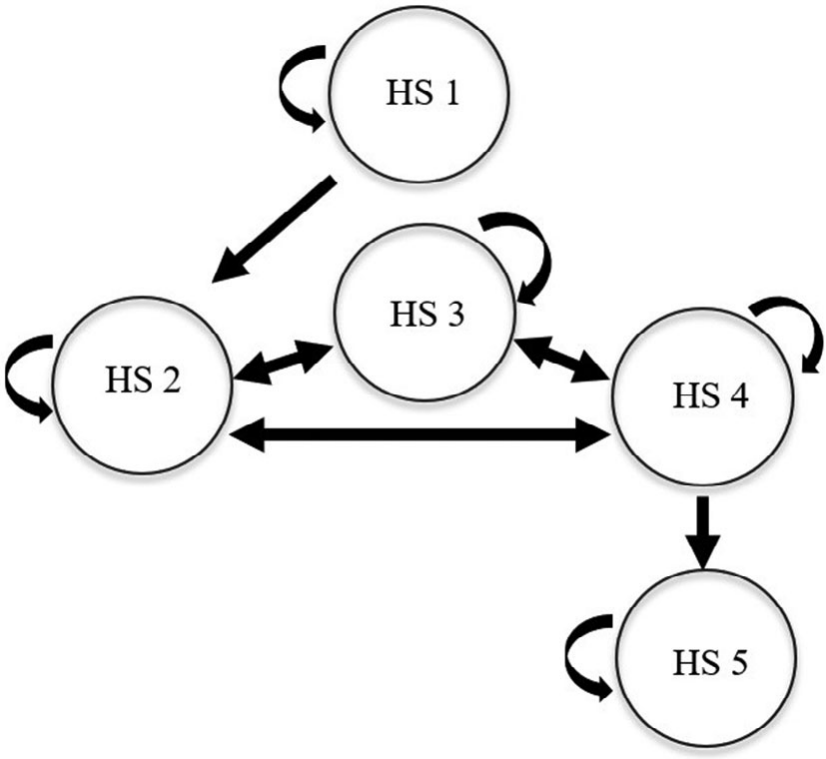
Markov model structure.

The Markov model consists of five health states based on the visual acuity of the better‐seeing eye. These states are based on vision acuity as classified by Finger et al. (2013), and they are presented in Table 1 and Figure 1. All patients start from health state one, in which only one eye has DMO and vision acuity is good, and health state five, in which the patient is blind, is the endpoint of the model. Once the treatment has started, it is continued during the whole follow‐up period, so there is no return to health state one. In addition, the model does not have a health state in which patients are cured completely, because the clinical evidence does not suggest that (e.g. Beck et al. 2009; Boyer et al. 2014).

**Table 1:** Markov model health states and their descriptions.

Health State	Description of the health state	Visual acuity of the better‐seeing eye (Snellen lines)*
1	One eye is healthy, and the other one has DMO. Vision acuity is good.	≥0.5
2	Both eyes have DMO. Visual acuity is good.	≥0.5
3	Both eyes have DMO. Mild visual impairment.	<0.5 but ≥0.3
4	Both eyes have DMO. Moderate to severe visual impairment.	<0.3 but ≥0.05
5	Both eyes have DMO. Blindness.	<0.05

* Vision acuity classified by Finger et al. (2013)

The perspective of this analysis is the hospital perspective. The societal perspective is not included in the analysis since the hospital makes the decisions about resource allocation.

### Population characteristics

A population of 1000 hypothetical patients was chosen for the cost‐effectiveness model. All of the patients are DM patients who have DMO in one eye, and their vision acuity is good (≥0.5). Based on cohort studies, this patient population could be described as follows: the starting age of DMO treatment is, on average, 66 years. Most of the patients are type 2 diabetics (93%), and 60% of them are men and 40% women. Phakic eyes account for 68% and pseudophakic for 32% of the patients (Ockrim et al. 2008; Fernández et al. 2010; Ramu et al. 2015; Malclés et al. 2017; Singer et al. 2018).

In the model, patients receive corticosteroid treatment throughout the follow‐up. Since the longest follow‐up period in the literature for corticosteroid treatment seems to be five years (e.g. Gillies et al. 2009), two different time horizons were used in the model: 2 and 5 years. No subgroup analyses were conducted in this analysis, as it is demonstrated that population characteristics, such as previous treatment, sex, pseudophakia/phakia, or vitrectomized/nonvitrectomized eyes, may not be relevant for corticosteroid treatment efficacy (Malclés et al. 2017; Cevik et al. 2018).

### Treatment strategies

The comparators included in the model are dexamethasone implants (OZURDEX 0.7 mg) and triamcinolone injections (TRIESENCE 40 mg/ml). Both treatments are proved to improve vision acuity significantly, but triamcinolone reduces macular thickness more effectively than dexamethasone (Dang et al. 2014; Schmidt‐Erfurt et al. 2017). As the cost of a dexamethasone implant (€1075) is far more that of a triamcinolone injection (€207), triamcinolone can be applied first in clinical practice.

The dosing regimen varies between the treatments, so the cycle length in the model also varies. A five‐month cycle was chosen for dexamethasone, since this is a common and often recommended re‐treatment schedule (e.g. Mathew et al., 2014; Bucolo et al. 2018). For triamcinolone, a cycle length of four months was chosen, as the maximum improvement in vision acuity occurs at three months, and after six months its effectiveness is no longer significant (Beer et al. 2003; Fernández et al. 2010; Qi et al. 2012; Jeon & Lee 2014). It is assumed that re‐treatment is not applied when the effectiveness is at its highest, but slightly after that. In addition, since it is known that triamcinolone requires re‐treatment sooner than dexamethasone (Dang et al. 2014), its cycle length must be shorter than that of dexamethasone. Cycle lengths are constant over the whole follow‐up period so that the model remains sufficiently simple.

### Disease progression

Transition probabilities were sourced from clinical, retrospective, and prospective studies. For both treatments, two transition matrices were made: one for a shorter follow‐up period (<12 months) and one for a longer follow‐up period (>12 months). The use of two transition matrices illustrates that the first doses are more effective than the subsequent doses (Chan et al. 2006). Transitions are based on changes in vision acuity, as a 10‐letter or 2‐line change can move a patient to the next health state and a 15‐letter or 3‐line change enables a patient to move more than one health state. Almost all transition probabilities are of several studies. Only studies with *n *> 50 are included in the calculations of transition probabilities. Studies used in the transition probabilities are available in the supplement (Sutter et al. 2004; Gillies et al. 2006; Thompson 2006; Özdek et al. 2006; Kuppermann et al. 2007; Lam et al. 2007; Ockrim et al. 2008; Beck et al. 2009; Fernandez et al. 2010; Haller et al. 2010; Boyer et al. 2011; Scanlon et al. 2013; Gillies et al. 2014; Lozano‐Lopez et al. 2015; Ramu et al. 2015; Callanan et al. 2017; Malclés et al. 2017; Pareja‐Rios et al. 2018; Singer et al. 2018).

When examining the Markov model of this cost‐effectiveness analysis, triamcinolone is slightly more effective at improving visual acuity than dexamethasone, and the re‐treatment schedule and occurrence of adverse events for triamcinolone are more frequent than for dexamethasone. In these points, the model is in line with existing direct comparisons.

### Cost and health outcome estimates

The direct medical costs included in the model are as follows:

1. the cost of the treatment, including the cost of the medicine and the cost of the administration
2. the cost of the follow‐up and diagnosis, including intraocular pressure measurement, fluorescein angiography (FAG), optical coherence tomography (OCT), and medical visits
3. the rehabilitation cost for legally blind persons

Corticosteroid treatment can cause adverse events, such as intraocular pressure (IOP) elevation, glaucoma, cataract, endophthalmitis, and retinal detachment. Triamcinolone has more adverse events than dexamethasone (Dang et al. 2014). The treatment of these adverse events is considered in the model. Cataract surgery and laser/vitrectomy due to IOP elevation are addressed only once during the whole follow‐up, whereas other adverse events can occur with every cycle. All unit costs are based on the costs in the Kuopio University Hospital price catalogue for 2019. The unit costs, utilization, and cost per year are presented in Tables 2 and 3.

**Table 2:** Costs in relation to the treatment.

	Unit cost		Utilization per year		Cost per year			
	Dexamethasone	Triamcinolone	Dexamethasone	Triamcinolone	Dexamethasone		Triamcinolone	
Cost of medication	€1075.00	€207.00	2.4	3	€2 580.00		€621.00	
Cost of administration								
Medical visit (DEX)	€90.00	‐	2.4	‐	€216.00		‐	
Nurse visit (TA)	‐	€76.00	‐	3	‐		€228.00	
Cost of follow‐up								
OCT + medical visit	€50.00 + €65.00	€50.00 + €65.00	6	6	€690.00		€690.00	
IOP measurement	€76.00	€76.00	13	13	€988.00		€988.00	
FAG	€77.00	€77.00	1	1	€77.00		€77.00	
					€1 755.00		€1 755.00	
						
Cost of adverse events *			Incidences per cycle					
IOP rise and glaucoma			Dexamethasone 5 months	Triamcinolone 4 months				
Medication: medication + medical visit**	€12.55 + €65.00	€12.55 + €65.00	8.90%	12.28%				
Laser***	€82.00	€82.00	0.49%	0.65%				
Vitrectomy***	€1195.00	€1195.00	0.47%	1.29%				
Cataract***	€1373.00	€1373.00	5.77%	12.64%				
Endophthalmitis**	€3101.00	€3101.00	0.25%	0.20%				
Retinal detachment**	€2706.00	€2706.00	0.04%	0.03%				

* Incidences of adverse events are based on the following studies: Boyer et al. (2014); Beck et al. (2009); Callanan et al. (2013); Ciardella et al. (2004); Fraser‐Bell et al. (2016); Gillies et al. (2006); Gillies et al. (2009); Haller et al. (2010); Maia et al. (2007); Sonmez & Ozturk (2012); Ramu et al. (2015); Malclés et al. (2017); Singer et al. (2018).

** Treatment of the adverse event is applied in every cycle during the follow‐up.

*** Treatment of the adverse event is applied only once during the follow‐up.

**Table 3:** Total costs of the treatment and the rehabilitation costs.

Total cost of the treatment per year	2‐year time horizon	5‐year time horizon
Dexamethasone	**€4 589.99**	**€4 479.02**
Triamcinolone	**€2 659.77**	**€2 639.93**
Rehabilitation costs*	**Cost per year**	
	Dexamethasone	Triamcinolone
Occupational rehabilitation	€2 496.00	€2 496.00
Discretionary rehabilitation	€2 176.00	€2 176.00
Medical rehabilitation	€2 648.00	€2 648.00
	€7 320.00	€7 320.00

* Source for the rehabilitation costs: Kelasto, 2018a,Kelasto, 2018b,Kelasto, 2018c.

If both eyes have DMO, both eyes are treated. This leads to double drug costs in states 2, 3, and 4. Rehabilitation costs due to visual impairment are applied in states 4 and 5. Utilities associated with health states are presented in Table 4 (Brown et al. 2000; Brown et al. 2001). Although these utilities are calculated for age‐related macular degeneration, they are also applicable for DMO (Brown et al. 1999). Discounting rates of 0% and 3% per annum were applied in the model for both costs and utilities.

**Table 4:** Health state utilities.

Health State	Utility
1	0.97
2	0.89
3	0.81
4	0.55
5	0.40

### Analysis

The incremental cost per incremental QALY was calculated as a final result of the model. Since there is uncertainty in the modelling, deterministic sensitivity analyses were performed. In the first sensitivity analysis, the cycle length for triamcinolone was changed from 4 months to 3 months. In the second sensitivity analysis, the cost of a dexamethasone implant was lowered by 20%. Increasing the cost of a dexamethasone implant was not investigated because is it rather unlikely that the price would rise. The third sensitivity analysis considered different utilities for health states. With these alternative utilities, the health state utility was higher in states 2, 4, and 5 and lower in health states 1 and 3 compared to the model. These alternative utilities were based on eye diseases in general (Sharma et al. 2000).

## Results

According to this cost‐effectiveness analysis, over a 2‐year horizon, gaining one additional discounted QALY would cost an additional discounted €56,591 when comparing dexamethasone to triamcinolone. Over a five‐year horizon, the discounted incremental cost‐effectiveness ratio is ‐€1 110 942 per QALY gained, so the result changes entirely when time horizon changes. Negative ICER means that triamcinolone is a dominant treatment strategy compared to dexamethasone over a 5‐year time horizon. The results of this cost‐effective analysis per 1000 patients are presented in Table 5.

**Table 5:** Results of Markov model analysis of two‐ and five‐year periods per 1,000 patients

2‐year time horizon			
Medication	Expected costs, €	Expected QALYs	ICER (€/QALY)*
0% discount rate			
Dexamethasone	9.810.196	2016.28	
Triamcinolone	5.562.866	1936.28	
Difference	4.247.330	80.00	53.089
3% discount rate			
Dexamethasone	9.454.583	1943.48	
Triamcinolone	5.374.726	1870.94	
Difference	4.079.857	72.54	56.243

5‐year time horizon			
Medication	Expected costs, €	Expected QALYs	ICER (€/QALY)
0% discount rate			
Dexamethasone	23.861.157	4821.46	
Triamcinolone	13.785.330	4825.50	
Difference	10.075.826	−4.04	−2.494.639
3% discount rate			
Dexamethasone	22.027.999	4455.23	
Triamcinolone	12.745.504	4464.36	
Difference	9.282.495	−9.13	−1.016.727

* The differences depicted here do not give the exact ICERs presented here due to rounding numbers.

Differences in the costs are due to differences in the prices of a dexamethasone implant and a triamcinolone injection. The cost of the treatment of adverse events did not affect the results significantly. Differences in the effectiveness (QALYs) are due to a small difference in the treatment efficacy, which is seen in the transition probabilities.

In the sensitivity analyses, we found that over a 2‐year time horizon, the results are sensitive to the cycle length of triamcinolone and to the cost of a dexamethasone implant. Using a different source for utilities had no significant impact on the results. Over a 5‐year time horizon, triamcinolone was dominant regardless of the sensitivity analyses. The sensitivity analyses are presented in Table 6.

**Table 6:** Discounted ICER values of the sensitivity analyses (discount rate 3%).

Sensitivity analysis	ICER (€/QALY)
2‐year time horizon	
3‐month cycle length for triamcinolone	50.880
The cost of a dexamethasone implant lowered by 20%	41.637
Alternative utilities (Sharma et al. 2000)*	58.678
5‐year time horizon	
3‐month cycle length for triamcinolone	−537.182
The cost of a dexamethasone implant lowered by 20%	−743.536
Alternative utilities (Sharma et al. 2000)*	−1.105.954

* Alternative utilities according to Sharma et al. (2000): State 1: 0.93; State 2: 0.85; State 3: 0.66; State 4: 0.58; State 5: 0.53, Calculated as an average of health state’s range.

## Discussion

This cost‐effectiveness analysis aimed to discover which of the corticosteroid treatments in use, triamcinolone or dexamethasone, is a cost‐effective treatment for DMO. The length of the follow‐up has a critical impact on whether triamcinolone or dexamethasone should be considered as cost‐effective. Both the 2‐ and 5‐year time horizons are important from the clinical point of view.

According to this cost‐effectiveness analysis, dexamethasone has bigger expected costs and better expected effectiveness than triamcinolone over a two‐year time horizon. The ICER is €56 591/QALY. Interpretation of the cost‐effectiveness depends on the decision‐maker’s willingness to pay (WTP) for better effectiveness. Considering the recommendations for the general WTP threshold (£20 000–30 000/QALY) (National Institute for Health & Care Excellence 2018), triamcinolone is cost‐effective compared to dexamethasone over a two‐year time horizon. In order to consider dexamethasone as cost‐effective, WTP should be around €55 000/QALY, which could be possible within the recommendations, since higher ICERs can be acceptable based on, for example, disease severity and the innovativeness of the treatment. A dexamethasone implant could meet these requirements since it is an innovative and unique treatment for DMO and preventing vision impairment overall is both clinically and economically important (Gonder et al. 2014; Kiss et al. 2016).

Over a 5‐year time horizon, the expected costs of dexamethasone are bigger than those of triamcinolone and, in addition, triamcinolone has better expected effectiveness than dexamethasone. Incremental cost‐effectiveness ratio (ICER) for a five‐year follow‐up is −€1 110 942/QALY. This means that over a 5‐year time horizon, triamcinolone is dominant compared to dexamethasone and it should be chosen over dexamethasone in clinical practice. This difference in results based on time horizon arises from the difference in the expected QALYs of dexamethasone and triamcinolone: the longer the time horizon, the better the effectiveness of triamcinolone over dexamethasone. Eventually, triamcinolone has more expected QALYs and lower expected costs compared to dexamethasone. This is due to the lower price of triamcinolone injections and their better efficacy, as seen in the transition probabilities. As triamcinolone causes more adverse events than dexamethasone, but their degenerative impact on quality of life is not considered in the model, triamcinolone seems to be more effective than dexamethasone.

In the sensitivity analyses, we found that the cycle length of triamcinolone and the unit cost of dexamethasone affects slightly the results. If the dosing interval of triamcinolone was three months instead of 4 months, ICER would be €51 408/QALY, which is somewhat lower than in the actual analysis. Again, if the unit cost of dexamethasone was 20% lower, ICER would be lower than in the actual analysis, being then €42 222/QALY. If the cycle length for triamcinolone was three months or the cost of the dexamethasone implant was 20% lower, dexamethasone could be accepted as cost‐effective compared to triamcinolone in the model over a two‐year time horizon with a WTP around €50 000/QALY and €40 000/QALY, respectively. Over a five‐year time horizon, triamcinolone would still be dominant compared to dexamethasone, regardless of the sensitivity analyses.

In the model, both eyes were included, which is one strength of this cost‐effectiveness analysis. Generally, one eye is defined as the better‐seeing eye (BSE) and the other one as the worse‐seeing eye (WSE). Cost‐effectiveness analyses made for eye diseases usually observe only one eye: the WSE and its vision and vision‐related quality of life. With this, the treatment effect on vision‐related quality of life is more remarkable than if both eyes were considered in the model. If the BSE was also considered, the treatment effect on vision‐related quality of life would be less, since the BSE has more effect on quality of life than the WSE (Hirneiss 2014). In order to obtain appropriate utility values, it is necessary to consider vision acuity in the other eye, too.

### Study limitations

When working with modelling, there are always uncertain factors that weaken the reliability of the analysis. Some of the uncertainties are due to the modelling itself; we cannot investigate a population at an individual level, rather we must settle for examining an average population. In reality, an average patient does not exist, so the modelling gives only an estimation for clinical practice. Simulation could enable individual‐level examination, but we did not have that kind of data available for this study. In addition, we must create some assumptions in the model in order to keep it sufficiently simple. These assumptions narrow our examination, which weakens its reliability. Sensitivity analyses are carried out to improve the reliability of this modelling.

One of the relevant assumptions applied to this model was that a patient cannot be completely cured of DMO, meaning that in the model transition, a return to health state one is impossible and there is no health state in which both eyes would be healthy. There was no evidence in clinical research of a patient being able to discontinue the treatment due to a desirable response, hence the assumption of incurable DMO. We also assumed that first doses of the treatment are more effective than the second ones, as this is demonstrated in the research literature (Chan et al. 2006). This is considered in the model when calculating the transition probabilities. In clinical practice, it is likely that the dosing interval becomes rarer over time (e.g. Malclés et al. 2017), but in this model the dosing regimen is fixed.

In this model, a health care perspective was chosen. It is the most common perspective regarding cost‐effectiveness analyses (Rawlins 2012) and relevant in Finland where hospital decides the medication. A societal perspective would have captured all the costs regardless of how they are allocated. These costs would include, for example, travel costs and the time costs of the caregivers who accompany the patient to the hospital. Considering that the corticosteroid treatment is received in hospital conditions and the dosing frequency is one of the main differences between these two treatments, it would have affected the result in favour of dexamethasone. It is likely that the final conclusion of the analysis would have remained unchanged: the 2‐year ICER would have lowered and the 5‐year dominance would still be present as adding new costs would not affect the difference in QALYs that was in favour of triamcinolone.

The societal perspective therefore presents a wider view on the consequences of health care interventions as the health care perspective gives more detailed information on resource implications for the health care decision makers (Neumann & Sanders 2017). As the decision maker in this case is the hospital, it was concluded sufficient to examine only the health care perspective in this analysis. From societal perspective, though, this approach may lead to partial optimization because the societal costs are not meaningful for a hospital to consider.

The transition probabilities were sourced from clinical (RCT), retrospective, and prospective studies. It is commonly recommended to use RCT studies for transition probabilities to ensure inner validity (Drummond et al. 2015), but since available RCT studies did not contain all the needed information for the transitions, and the follow‐up period in them was rather short, we had to complement them with other studies. There are only a few clinical studies that compare dexamethasone and triamcinolone directly. In most of the chosen studies, the comparator was a placebo or there was no comparator at all, so the comparison in this cost‐effectiveness analysis was somewhat indirect. Since there is no single study that includes all the necessary information for this cost‐effectiveness analysis, several studies were required, and the averages of their results were used.

It is worth considering whether a degenerative impact on quality of life as a consequence of adverse events should be included in the model. Quality of life degenerates as vision acuity decreases (e.g. Brown et al. 1999; Sharma et al. 2000), which is considered within the model itself. Adverse events are incorporated into the model only by considering the costs of their treatment. However, because adverse events cause other not vision‐related inconveniences to patients (e.g. pain, anxiety, and loss of time), the quality of life decrements caused by adverse events could be worth examining.

## Conclusions

In this cost‐effectiveness analysis, we found that triamcinolone was cost‐effective compared to dexamethasone over both 2‐ and 4‐year time horizons, if the WTP threshold is considered as €30 000/QALY. Over a 5‐year time horizon, triamcinolone was also a dominant treatment strategy. However, over a two‐year time horizon, it could be possible for a decision maker to accept dexamethasone as cost‐effective compared to triamcinolone if the WTP was around €55 000/QALY. A threshold of €55 000/QALY for WTP could be possible, since DMO itself has a great burden of illness, and a dexamethasone implant can be seen as an innovative approach for treating DMO. In addition, the sensitivity analyses support the cost‐effectiveness of dexamethasone over the two‐year time horizon with the threshold for WTP around €50 000/QALY.

Even if a decision‐maker accepted dexamethasone as a cost‐effective treatment, however, this does not mean that it would be possible to use it in practice. Both WTP and available budget have an impact on whether a new drug can be introduced in clinical practice or not. It is worth noting that the cost per annum of dexamethasone is 66% higher compared to triamcinolone, so it would require a lot more resources in order to choose to use dexamethasone over triamcinolone. Therefore, a cost‐effectiveness analysis alone cannot give an adequate recommendation about resource allocation.

In the guidelines for the management of diabetic macular oedema by the European society of Retina Specialists (EURETINA), it is recommended to apply dexamethasone first (Schmidt‐Erfurt et al. 2017), and therefore, triamcinolone should be used only if dexamethasone does not give the desired response. Given that a dexamethasone implant is an innovative treatment, the WTP threshold could be around €55 000/QALY, meaning that according to this analysis, dexamethasone would be cost‐effective over a two‐year time horizon. After that, triamcinolone would explicitly be a reasonable choice for DMO treatment. Thus, this cost‐effectiveness analysis does not necessarily require changes in the current recommendation. However, it can be argued whether or not the recommendation is sensible, since the difference in the prices of a triamcinolone injection and a dexamethasone implant is so significant. Therefore, the budget impact must also be considered when allocating scarce resources. Moreover, there is no single threshold for WTP that would be appropriate for all decisions, so the conclusion for the cost‐effectiveness has aspects that this cost‐effectiveness analysis cannot solely resolve.

## References

[aos14745-bib-0001] Al Dhibi, HA & Arevalo, F (2013): Clinical trials on corticosteroids for diabetic macular edema. World Journal of Diabetes, 4(6): 295–302. 24379920 10.4239/wjd.v4.i6.295 PMC3874489

[aos14745-bib-0002] American Diabetes Association (2019): Standards of medical care in diabetes ‐ 2019 abridged for primary care providers. Clin Diabetes 37(1): 11–34. 30705493 10.2337/cd18-0105 PMC6336119

[aos14745-bib-0003] Beck RW , Edwards AR , Aiello LP et al. (2009): Three‐year follow‐up of a randomized trial comparing focal/grid photocoagulation and intravitreal triamcinolone for diabetic macular edema. Arch Ophthalmol 127(3): 245–251. 19273785 10.1001/archophthalmol.2008.610 PMC2754047

[aos14745-bib-0004] Beer PM , Bakri SJ , Singh RJ , Liu W , Peters GB & Miller M (2003): Intraocular concentration and pharmacokinetics of triamcinolone acetonide after a single intravitreal injection. Ophthalmology 110(4): 681–686. 12689886 10.1016/S0161-6420(02)01969-3

[aos14745-bib-0005] Boyer DS , Faber D , Gupta S et al. (2011): Dexamethasone intravitreal implant for treatment of diabetic macular edema in vitrectomized patients. Retina 31(5): 915–923. 21487341 10.1097/IAE.0b013e318206d18c

[aos14745-bib-0006] Boyer DS , Yoon YH , Belfort R et al. (2014): Three‐year, randomized, sham‐controlled trial of dexamethasone intravitreal implant in patients with diabetic macular edema. Ophthalmology 121: 1904–1914. 24907062 10.1016/j.ophtha.2014.04.024

[aos14745-bib-0007] Brown GC (1999): Vision and quality‐of‐life. Trans Am Ophthalmol Soc 97: 473–511. 10703139 PMC1298275

[aos14745-bib-0008] Brown GC , Sharma S , Brown MM & Kistler J (2000): Utility values and age‐related macular degeneration. Am Med Assoc Arch Ophthalmol 118(1): 47–51. 10.1001/archopht.118.1.47 10636413

[aos14745-bib-0009] Brown MM , Brown GC , Sharma S , Busbee B & Brown H (2001): Quality of life associated with unilateral and bilateral good vision. Ophthalmology 108(4): 643–648. 11297474 10.1016/s0161-6420(00)00635-7

[aos14745-bib-0010] Bucolo C , Gozzo L , Longo L , Mansueto S , Vitale DC & Drago F (2018): Long‐term efficacy and safety profile of multiple injections of intravitreal dexamethasone implant to manage diabetic macular edema: A systematic review of real‐world studies. J Pharm Sci 138(4): 219–232. 10.1016/j.jphs.2018.11.001 30503676

[aos14745-bib-0011] Busch C , Zur D , Fraser‐Bell S et al. (2018): Shall we stay, or shall we switch? Continued anti‐VEGF therapy versus early switch to dexamethasone implant in refractory diabetic macular edema. Acta Diabetol 55(8): 789–796. 29730822 10.1007/s00592-018-1151-x

[aos14745-bib-0012] Callanan DG , Gupta S , Boyer DS et al. (2013): Dexamethasone intravitreal implant in combination with laser photocoagulation for the treatment of diffuse diabetic macular edema. Ophthalmology 120(9): 1843–1851. 23706947 10.1016/j.ophtha.2013.02.018

[aos14745-bib-0013] Callanan DG , Loewenstein A , Patel SS et al. (2017): A multicenter, 12‐month randomized study comparing with diabetic macular edema dexamethasone intravitreal implant with ranibizumab in patients with diabetic macular edema. Graefes Arch Clin Exp Ophthalmol 255(3): 463–473. 27632215 10.1007/s00417-016-3472-1

[aos14745-bib-0014] Cevik SG , Yilmaz S , Cevik MT , Akalp FD & Avci R . (2018): Comparison of the effect of intravitreal dexamethasone implant in vitrectomized and nonvitrectomized eyes for the treatment of diabetic macular edema. J Opthalmol 2018: 1–8. 10.1155/2018/1757494 PMC5937369 29850199

[aos14745-bib-0015] Chan CKM , Mohamed S , Shanmugam MP , Tsang C‐W , Lai TYY & Lam DSC (2006): Decreasing efficacy of repeated intravitreal triamcinolone injections in diabetic macular oedema. Br J Ophthalmol 90(9): 1137–1141. 16707525 10.1136/bjo.2006.093211 PMC1857409

[aos14745-bib-0016] Chang‐Lin JE , Attar M , Acheampong AA , Robinson MR , Whitcup SM , Kuppermann BD & Welty D (2011): Pharmacokinetics and pharmacodynamics of a sustained‐release dexamethasone intravitreal implant. The association for research in vision and ophthalmology. Invest Ophthalmol Vis Sci 52(1): 80–86. 20702826 10.1167/iovs.10-5285

[aos14745-bib-0017] Ciardella AP , Klancnik J , Schiff W , Barile G , Langton K & Chang S (2004): Intravitreal triamcinolone for the treatment of refractory diabetic macular oedema with hard exudates: an optical coherence tomography study. Br J Ophthalmol 88(9): 1131–1136. 15317702 10.1136/bjo.2004.041707 PMC1772301

[aos14745-bib-0018] Dang Y , Mu Y , Li L , Mu Y , Liu S , Zhang C , Zhu Y & Xu Y (2014): Comparison of dexamethasone intravitreal implant and intravitreal triamcinolone acetonide for the treatment of pseudophakic cystoid macular edema in diabetic patients. Drug Design Dev Ther 8: 1441–1449. 10.2147/DDDT.S66611 PMC4174031 25258512

[aos14745-bib-0019] Dewan V , Lambert D , Edler J , Kymes S & Apte RS (2012): Cost‐effectiveness analysis of ranibizumab plus prompt or deferred laser or triamcinolone plus prompt laser for diabetic macular edema. Ophthalmology 119(8): 1679–1684. 22503301 10.1016/j.ophtha.2012.01.049 PMC3612959

[aos14745-bib-0020] Drummond MF , Sculpher MJ , Claxton K , Stoddart GL & Torrance GW (2015): Methods for the Economic Evaluation of Health Care Programmes. Oxford: Oxford University Press, pp. 332–334.

[aos14745-bib-0021] Duh EJ , Sun JK & Stitt AW (2017): Diabetic retinopathy: current understanding, mechanisms, and treatment strategies. American Society for Clinical Investigation. JCI. Insight 2(14): 1–13. 10.1172/jci.insight.93751 PMC5518557 28724805

[aos14745-bib-0022] Fernández G , Alonso G , Gil F & Villa R (2010): Intravitreal triamcinolone acetonide use in differuse persistent diabetic macular edema. Arch Soc Española Oftalmol 86(10): 314–319. 10.1016/j.oftal.2011.05.018 22004576

[aos14745-bib-0023] Finger RP , Fenwick E , Hirneiss CW , Hsueh A , Guymer RH , Lamoureux EL & Keeffe JE (2013): Visual impairment as a function of visual acuity in both eyes and its impact on patient reported preferences. PLoS One 8(12): e81042. 24339893 10.1371/journal.pone.0081042 PMC3855212

[aos14745-bib-0024] Finnish Medical Agency 2019. Lääkehaut ja luettelot. Fimea Web, Iluvien. (13.4.2020).

[aos14745-bib-0025] Ford JA , Clar C , Lois N , Barton S , Thomas S , Court R , Shyangdan D & Waugh N (2014): Treatments for macular oedema following central retinal vein occlusion: systematic review. BMJ Open 4(2): 1–11. 10.1136/bmjopen-2013-004120 PMC3927713 24513867

[aos14745-bib-0026] Fraser‐Bell S , Lim LL , Campain A et al. (2016): Bevacizumab or dexamethasone implants for DME: 2‐year results (The BEVORDEX Study). Ophthalmology 123(6): 1399–1401. 26783096 10.1016/j.ophtha.2015.12.012

[aos14745-bib-0027] Gillies MC , Lim LL , Campain A et al. (2014): A randomized clinical trial of intravitreal bevacizumab versus intravitreal dexamethasone for diabetic macular edema. The BEVORDEX study. Ophthalmology 121(12): 2473–2481. 25155371 10.1016/j.ophtha.2014.07.002

[aos14745-bib-0028] Gillies MC , Simpson JM , Gaston C , Hunt G , Ali H , Zhu M & Sutter F (2009): Five‐year results of a randomized trial with open‐label extension of triamcinolone acetonide for refractory diabetic macular edema. Ophthalmology 116(11): 2182–2187. 19796823 10.1016/j.ophtha.2009.04.049

[aos14745-bib-0029] Gillies MC , Sutter FKP , Simpson JM , Larsson J , Ali H & Zhu M (2006): Intravitreal triamcinolone for refractory diabetic macular edema: two‐year results of a double‐masked, placebo‐controlled, randomized clinical trial. Ophthalmology 113(9): 1533–1538. 16828501 10.1016/j.ophtha.2006.02.065

[aos14745-bib-0030] Gonder JR , Walker VM , Barbeau M , Zaour N & Zachau BH , Hartje JR & Li R (2014): Costs and quality of life in diabetic macular edema: canadian burden of diabetic macular edema observational study (C‐REALITY). J Ophthalmol 2014: 1–9. 10.1155/2014/939315 PMC3984851 24795818

[aos14745-bib-0031] Guariguata L , Whiting DR , Hambleton I , Beagley J , Linnenkamp U & Shaw JE (2014): Global estimates of diabetes prevalence for 2013 and projections for 2035. Diabetes Res Clin Pract 103(2): 137–149. 24630390 10.1016/j.diabres.2013.11.002

[aos14745-bib-0032] Haller JA , Kuppermann BD , Blumenkranz MS , Williams GA , Weinberg DV , Chou C & Whitcup SM (2010): Randomized controlled trial of an intravitreous dexamethasone drug delivery system in patients with diabetic macular edema. Arch Ophthalmol 128(3): 289–296. 20212197 10.1001/archophthalmol.2010.21

[aos14745-bib-0033] Hirneiss C (2014): The impact of a better‐seeing eye and a worseseeing eye on vision‐related quality of life. Clin Ophthalmol 8: 1703–1709. 25214763 10.2147/OPTH.S64200 PMC4159393

[aos14745-bib-0034] Jeon S & Lee WK (2014): Effect of intravitreal triamcinolone in diabetic macular edema unresponsive to intravitreal bevacizumab. Retina 34(8): 1606–1611. 24553409 10.1097/IAE.0000000000000109

[aos14745-bib-0035] Kelasto (2018a). Kelan kuntoutuspalvelujen saajat ja kustannukset. Koko maa. Vuodet 2014–2018. Ammatillinen kuntoutus. Silmän ja sen apuelinten sairaudet. (9.4.2020).

[aos14745-bib-0036] Kelasto (2018b). Kelan kuntoutuspalvelujen saajat ja kustannukset. Koko maa. Vuodet 2014–2018. Harkinnanvarainen kuntoutus. Silmän ja sen apuelinten sairaudet. (9.4.2020).

[aos14745-bib-0037] Kelasto (2018c). Kelan kuntoutuspalvelujen saajat ja kustannukset. Koko maa. Vuodet 2014–2018. Vaativa lääkinnällinen kuntoutus. Silmän ja sen apuelinten sairaudet. (9.4.2020).

[aos14745-bib-0038] Kiddee, W , Trope, GE , Sheng, L , Beltran‐Agullo, L , Smith, M , Strungar, MH , Baath, J & Buys, YM (2013): Intraocular Pressure Monitoring Post Intravitreal Steroids: A Systematic Review. Survey of Ophthalmology, 58(4): 291–310. 23768920 10.1016/j.survophthal.2012.08.003

[aos14745-bib-0039] Kiss S , Chandwani HS , Cole AL , Patel VD , Lunacsek OE & Dugel PU (2016): Comorbidity and health care visit burden in working‐age commercially insured patients with diabetic macular edema. Clin Ophthalmol 10. 10.2147/OPTH.S114006 PMC5153291 27994438

[aos14745-bib-0040] Kuopio University Hospital . (2019). Kliinisten erikoisalojen palvelutuotteet, suoritteet ja hinnat. Talousjohtajan päätös 31§/19.12.2018. https://www.psshp.fi/documents/7796350/7869509/Klinikkahinnasto+2019.pdf/a45a6f1a‐97ce‐465d‐b65f‐ff4fdeda6795.

[aos14745-bib-0041] Kuppermann BD , Blumenkranz MS , Haller JA , Williams GA , Weinberg DV , Chou C & Whitcup SM (2007): Randomized controlled study of an intravitreous dexamethasone drug delivery system in patients with persistent macular edema. Arch Ophthalmol 125(3): 309–317. 17353400 10.1001/archopht.125.3.309

[aos14745-bib-0042] Lam DSC , Chan CKM , Mohamed S et al. (2007): A prospective randomised trial of different doses of intravitreal triamcinolone for diabetic macular oedema. Br J Ophthalmol 91(2): 199–203. 16973659 10.1136/bjo.2006.102848 PMC1857621

[aos14745-bib-0043] Lazic R , Lukic M , Boras I , Draca N , Vlasic M , Gabric N & Tomic Z (2014): Treatment of anti‐vascular endothelial growth factor‐resistant diabetic macular edema with dexamethasone invitreal implant. Retina 34(4). 10.1097/IAE.0b013e3182a48958 23975006

[aos14745-bib-0044] Leasher JL , Bourne RRA , Flaxman SR et al. (2016): Global estimates on the number of people blind or visually impaired by diabetic retinopathy: a meta‐analysis from 1990 to 2010. Diabetes Care 39(9): 1643–1649. 27555623 10.2337/dc15-2171

[aos14745-bib-0045] Liew G , Wong VW & Ho I (2017): Mini review: changes in the incidence of and progression to proliferative and sight‐threatening diabetic retinopathy over the last 30 years. Ophthal Epidemiol 24(2): 73–80. 10.1080/09286586.2016.1259638 28102748

[aos14745-bib-0046] Lozano López V , Serrano García M , Mantolán Sarmiento C et al. (2015): Resultados coste‐efectividad del implante de dexametasona en edema macular. Arch Soc Espanola Oftalmol 90(1): 14–21. 10.1016/j.oftal.2013.10.007 25443181

[aos14745-bib-0047] Maia M , Farah ME , Belfort RN , Penha FM , Lima F , Acacio AS , Aggio FB & Belfort R (2007): Effects of intravitreal triamcinolone acetonide injection with and without preservative. Br J Ophthalmol 91(9): 1122–1124. 17383993 10.1136/bjo.2007.115386 PMC1954894

[aos14745-bib-0048] Malclès A , Dot C , Voirin N , Agard É , Vié A‐L , Bellocq D , Denis P & Kodjikian L (2017): Real‐life study in diabetic macular edema treated with dexamethasone implant. The Reldex Study. Retina 37(4): 753–760. 27471826 10.1097/IAE.0000000000001234

[aos14745-bib-0049] Maniadakis N & Konstantakopoulou E (2019): Cost effectiveness of treatments for diabetic retinopathy: a systematic literature review. Pharmacoeconomics 37(8): 995–1010. 31012025 10.1007/s40273-019-00800-w

[aos14745-bib-0050] Mathew R , Pearce E , Muniraju R , Abdul‐Hey A & Sivaprasad S (2014): Monthly OCT monitoring of Ozurdex for macular oedema related to retinal vascular diseases: re‐treatment strategy (OCTOME Report 1). Eye 28(3): 318–326. 24384961 10.1038/eye.2013.287 PMC3965819

[aos14745-bib-0051] Maturi RK , Glassman AR , Liu D et al. (2018): Effect of adding dexamethasone to continued ranibizumab treatment in patients with persistent diabetic macular edema. A DRCR network phase 2 randomized clinical trial. JAMA Ophthalmology 136(1): 29–38. 29127949 10.1001/jamaophthalmol.2017.4914 PMC5833605

[aos14745-bib-0052] Mehta H , Hennings C , Gillies MC , Nguyen V , Campain A & Fraser‐Bell S . (2018): Anti‐vascular endothelial growth factor combined with intravitreal steroids for diabetic macular oedema. Cochr Database Syst Rev 4. 10.1002/14651858.CD011599.pub2 PMC6494419 29669176

[aos14745-bib-0053] Miller K & Fortun JA (2018): Diabetic macular edema: current understanding, pharmacologic treatment options, and developing therapies. Asia Pac J Ophthalmol 7(1): 28–35. 10.22608/APO.2017529 29473719

[aos14745-bib-0054] Mylonas G , Georgopoulos M , Malamos P et al. (2017): Comparison of dexamethasone intravitreal implant with conventional triamcinolone in patients with postoperative cystoid macular edema. Curr Eye Res 42(4): 648–652. 27612922 10.1080/02713683.2016.1214968

[aos14745-bib-0055] National Institute for Health and Care Excellence (2018): Guide to the processes of technology appraisal. 27905710

[aos14745-bib-0056] Neumann PJ & Sanders GD (2017): Cost‐effectiveness analysis 2.0. N Engl J Med 376(3): 203–205. 28099837 10.1056/NEJMp1612619

[aos14745-bib-0057] Ockrim ZK , Sivaprasad S , Falk S , Roghani S , Bunce C , Gregor Z & Hykin P (2008): Intravitreal triamcinolone versus laser photocoagulation for persistent diabetic macular oedema. Br J Ophthalmol 92(6): 795. 18420749 10.1136/bjo.2007.131771

[aos14745-bib-0058] Ojamo M (2018): Näkövammarekisterin vuosikirja 2017. Näkövammaisten liitto ry: Näkövammarekisteri.

[aos14745-bib-0059] Özdek S , Bahceci UA , Gürelik G & Hasanreisoglu B (2006): Posterior subtenon and intravitreal triamcinolone acetonide for diabetic macular edema. J Diabetes Its Complications 20(4): 246–251. 10.1016/j.jdiacomp.2005.06.015 16798476

[aos14745-bib-0060] Pareja‐Ríos A , Ruiz‐de la Fuente‐Rodríguez P , Bonaque‐González S , López‐Gálvez M , Lozano‐López V & Romero‐Aroca P (2018): Intravitreal dexamethasone implants for diabetic macular edema. International. J Ophthalmol 11(1): 77–82. 10.18240/ijo.2018.01.14 PMC5767662 29375995

[aos14745-bib-0061] Pershing S , Enns EA , Matesic B , Owens DK & Goldhaber‐Fiebert JD (2014): Cost‐effectiveness of treatment of diabetic macular edema. Ann Intern Med 160(1): 18–29. 24573663 10.7326/M13-0768 PMC4020006

[aos14745-bib-0062] Pochopien M , Beiderbeck A , McEwan P , Zur R , Toumi M & Aballéa S (2019): Cost‐effectiveness of fluocinolone acetonide implant (ILUVIEN®) in UK patients with chronic diabetic macular oedema considered insufficiently responsive to available therapies. BMC Health Serv Res 19(1). 10.1186/s12913-018-3804-4 PMC6327492 30626376

[aos14745-bib-0063] Qi HP , Bi S , Wei SQ , Cui H & Zhao JB (2012): Intravitreal versus subtenon triamcinolone acetonide injection for diabetic macular edema: a systematic review and meta‐analysis. Curr Eye Res 37(12): 1136–1147. 22793880 10.3109/02713683.2012.705412

[aos14745-bib-0064] Rajendran A & Badole P (2018): DME management – current perspective and therapeutic strategies. J Ophthalmol Related Sci 2(1): 7–14.

[aos14745-bib-0065] Ramu J , Yang Y , Menon G et al. (2015): A randomized clinical trial comparing fixed vs pro‐renata dosing of Ozurdex in refractory diabetic macular oedema (OZDRY study). Eye 29: 1603–1612. 26493038 10.1038/eye.2015.214 PMC5129797

[aos14745-bib-0066] Rawlins MD (2012): Crossing the fourth hurdle. Br J Clin Pharmacol 73(6): 855–860. 22404227 10.1111/j.1365-2125.2012.04263.x PMC3391507

[aos14745-bib-0067] Scanlon PH , Stratton IM , Histed M , Chave SJ & Aldington SJ (2013): The influence of background diabetic retinopathy in the second eye on rates of progression of diabetic retinopathy between 2005 and 2010. Acta Ophthalmol 91(5): 335–339. 23551550 10.1111/aos.12074 PMC3798105

[aos14745-bib-0068] Schmidt‐Erfurth U , Garcia‐Arumi J , Bandello F et al. (2017): Guidelines for the management of diabetic macular edema by the European Society of Retina Specialists (EURETINA). Ophthalmologica 237(4): 185–222. 28423385 10.1159/000458539

[aos14745-bib-0069] Shah AR , Yonekawa Y , Todorich B , Laere LV , Hussain R , Woodward MA , Abbey AM & Wolfe JD (2017): Prediction of Anti‐VEGF response in diabetic macular edema after 1 injection. J VitreoRetinal Dis 1(3): 169–174. 10.1177/2474126416682569 PMC5668872 29104958

[aos14745-bib-0070] Sharma S , Brown GC , Brown MM , Shah GK , Snow K , Brown H & Hollands H (2000): Converting visual acuity to utilities. Can J Ophthalmol 35(5): 267–272. 10959467 10.1016/s0008-4182(00)80077-0

[aos14745-bib-0071] Shin YU , Hong EH , Lim HW , Kang MH , Seong M & Cho H (2017): Quantitative evaluation of hard exudates in diabetic macular edema after short‐term intravitreal triamcinolone, dexamethasone implant or bevacizumab injections. BMC Ophthalmol 17(1). 10.1186/s12886-017-0578-0 PMC5627478 28974211

[aos14745-bib-0072] Singer MA , Dugel PU , Fine HF , Capone A & Maltman J (2018): Real‐world assessment of dexamethasone intravitreal implant in dme: findings of the prospective, multicenter REINFORCE study. Ophthal Surg Lasers Imaging Retina 49(6): 425–435. 10.3928/23258160-20180601-07 29927470

[aos14745-bib-0073] Smiddy WE (2011): Economic considerations of macular edema therapies. Ophthalmology (Rochester, Minn.) 118(9): 1827–1833. 10.1016/j.ophtha.2010.12.034 PMC3483086 21507488

[aos14745-bib-0074] Sonmez K & Ozturk F (2012): Complications of intravitreal triamcinolone acetonide for macular edema and predictive factors for intraocular pressure elevation. Int J Ophthalmol 5(6): 719–725. 23275907 10.3980/j.issn.2222-3959.2012.06.13 PMC3530815

[aos14745-bib-0075] Stewart M (2012): Corticosteroid use for diabetic macular edema: old fad or new trend? Curr Diab Rep 12(4): 364–375. 22581206 10.1007/s11892-012-0281-8

[aos14745-bib-0076] Sutter FKP , Simpson JM & Gillies MC (2004): Intravitreal triamcinolone for diabetic macular edema that persists after laser treatment. Three‐month efficacy and safety results of a prospective, randomized, double‐masked, placebo‐controlled clinical trial. Ophthalmology 111(11): 2044–2049. 15522370 10.1016/j.ophtha.2004.05.025

[aos14745-bib-0077] Thompson JT (2006): Cataract formation and other complications of intravitreal triamcinolone for macular edema. Am J Ophthalmol 141(4): 629–637. 16564796 10.1016/j.ajo.2005.11.050

[aos14745-bib-0078] Virgili G , Parravano M , Evans JR , Gordon I & Lucenteforte E (2018): Anti‐vascular endothelial growth factor for diabetic macular oedema: a network meta‐analysis (Review). Cochr Database Syst Rev 10. 10.1002/14651858.CD007419.pub6 PMC6517135 30325017

[aos14745-bib-0079] Zheng Y , He M & Condong N (2012): The worldwide epidemic of diabetic retinopathy. Indian J Ophthalmol 60(5): 428. 22944754 10.4103/0301-4738.100542 PMC3491270

